# Direct Measurement of the Stall Torque of the Flagellar Motor in Escherichia coli with Magnetic Tweezers

**DOI:** 10.1128/mbio.00782-22

**Published:** 2022-06-14

**Authors:** Bin Wang, Guanhua Yue, Rongjing Zhang, Junhua Yuan

**Affiliations:** a Department of Physics, University of Science and Technology of China, Hefei, Anhui, China; b School of Life Sciences, Zhengzhou University, Zhengzhou, China; University of Utah

**Keywords:** bacterial motility, magnetic tweezers, stall torque, tight coupling

## Abstract

The flagellar motor drives the rotation of flagellar filaments, propelling the swimming of flagellated bacteria. The maximum torque the motor generates, the stall torque, is a key characteristic of the motor function. Direct measurements of the stall torque carried out 3 decades ago suffered from large experimental uncertainties, and subsequently there were only indirect measurements. Here, we applied magnetic tweezers to directly measure the stall torque in E. coli. We precisely calibrated the torsional stiffness of the magnetic tweezers and performed motor resurrection experiments at stall, accomplishing a precise determination of the stall torque per torque-generating unit (stator unit). From our measurements, each stator passes 2 protons per step, indicating a tight coupling between motor rotation and proton flux.

## INTRODUCTION

The flagellar rotary motor in Escherichia coli converts transmembrane proton flux into flagellar rotation, propelling the swimming of bacteria. A motor torque-generating unit (a stator unit) is composed of five MotA and two MotB proteins, forming two proton-conducting transmembrane channels (1, 2). Driven by a proton electrochemical potential difference across the cytoplasmic membrane (the proton motive force, PMF), protonation and deprotonation of Asp32 in MotB at the cytoplasmic end of either channel induce conformational changes of a stator unit, which exerts force on the periphery of the rotor via electrostatic and steric interaction (3, 4), and the resulting torque is transmitted to the flagellar filament via a series of molecular shafts composed of a rod and a flexible hook. A motor can contain up to 11 functionally independent stators, exchanging with a membrane pool of stators on a timescale of 1 min (5–13).

A key property of the flagellar motor is its torque-speed relationship, measuring how much torque it generates at different speeds. This relationship was measured earlier with the electro-rotation method to vary the external torque (14, 15) and, subsequently, by labeling different sizes of latex beads to shortened filament stub or by changing medium viscosity to vary the viscous load (16–20). The motor torque is maximum at stall and stays approximately constant up to a knee speed, after which it drops rapidly to zero. In E. coli at room temperature, the knee speed is about 170 Hz, and the speed at zero torque is about 300 Hz. The stall torque per stator is one of the key characteristics of the flagellar motor. As the motor is in equilibrium at stall, one can infer how many protons a stator passes per revolution from the value of the stall torque and the PMF.

The earliest direct measurement of the stall torque for a wild-type flagellar motor was performed by flowing medium to stall the tethered cell, giving a value in the range of 1,000 to 5,000 pN×nm due to large experimental uncertainty (21). A subsequent measurement was conducted with optics tweezers, resulting in a value of about 4,500 pN×nm (22). As we were not able to determine the number of stators in a wild-type motor in those experiments, the number was usually assumed to be about 8, resulting in a value of stall torque per stator in the range of 125 to 625 pN×nm or about 563 pN×nm. Subsequently, indirect measurements were performed by labeling 1.0-μm-diameter beads to shortened filament stub and assuming that the torque under this high load is the same as the stall torque. Those indirect measurements generated a value of the stall torque per stator in the range of 146 to 320 pN×nm (6, 17, 23). The most recent indirect measurement with a sodium-driven chimeric motor in E. coli resulted in an estimate of each stator passing about 37 ions per revolution, inconsistent with the value of 26 or 52 ions as each motor takes 26 steps per revolution (24–26). This promoted the proposal of the mechanism of loose coupling between the proton flux and the motor rotation (27), in direct contrast to the long-held view that the proton flux and the motor rotation are tightly coupled (28–30).

Here, to resolve these inconsistencies, we applied magnetic tweezers to perform motor resurrection experiments at stall, so that we can directly measure both the stall torque and the stator number, resulting in a precise determination of the stall torque per stator.

## RESULTS

### Motor resurrection at stall.

**FIG 1:**
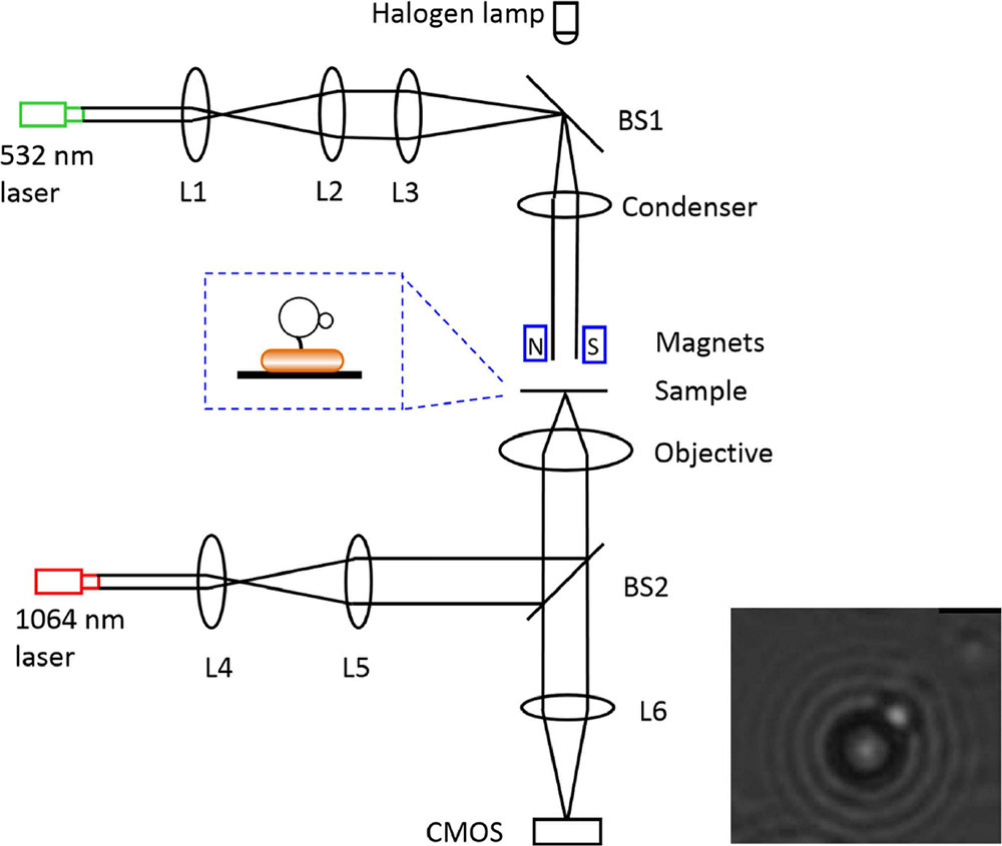
The experimental setup (see Materials and Methods for details). L1, L2, L3, L4, L5, and L6 are convex lenses, BS1 and BS2 are dichroic mirrors, N and S are the magnets for the magnetic tweezer, the 532-nm laser provides the light for exciting proteorhodopsin, the 1064-nm laser provides the light for the optic trap, the halogen lamp provides the light for bright-field imaging, and CMOS is the camera for bright-field imaging. The inset is a sketch of the sample. An example of a bright-field image of the beads is shown on bottom right.

A schematic of the experimental setup is presented in Fig. 1 (see details in Materials and Methods). Two permanent magnets generate the magnetic field for the tweezers. A magnetic bead was attached to the hook of the motor. If the motor was pulled to stall by the magnetic tweezers, motor torque was balanced by externally applied torque:

(1) $$T_{\mathrm{stall}}+k<\theta >=0,$$

where *T_stall_* is the stall torque of motors, *k* is the torsional stiffness of magnetic tweezers, and <*θ*> is the angular change for the orientation of the magnetic bead relative to that when the motor torque was zero. *k* depends on the magnitude of the magnetic field and the number and alignment of magnetic nano-particles in individual magnetic beads (31). The linearity between the torque of the magnetic tweezers and the angular change was verified by experiments previously (32) (see Text S1 in the supplemental material for details). *k* was calibrated by measuring the rotational thermal fluctuations of the bead orientation <*δθ*^2^> and applying the equipartition theorem *k* = *k_B_T*/<*δθ*^2^>. In practice, there were apparent differences among individual beads, so it was necessary to calibrate the torsional stiffness for each bead attached to a deenergized flagellar motor. The motor was then energized to generate torque.

10.1128/mbio.00782-22.1 TEXT S1 Power spectrum for the rotation of the magnetic bead in the magnetic tweezers. Linearity of the magnetic tweezer. Download Text S1, DOCX file, 0.02 MB. Copyright © 2022 Wang et al. 2022 Wang et al. https://creativecommons.org/licenses/by/4.0/ This content is distributed under the terms of the Creative Commons Attribution 4.0 International license.

The E. coli K-12 strain JY9, which was deleted for the genes *cheY* (so that the motor only rotates counterclockwise) and *fliC* (the filament gene) and carried mutated hook *flgE* expressing the hook protein with a tetracysteine motif, was transformed with the plasmid pTrc99aPR, which expresses the light-driven proton pump proteorhodopsin. The hook of the motor was biotinylated, and a streptavidin-coated 2.8-μm-diameter magnetic bead was labeled to the hook in motility buffer containing 23 mM NaN_3_ (to deenergize the motor). Then a 1-μm-diameter biotinylated bead was manipulated with an optical trap to attach to the magnetic bead as a fiducial marker for characterizing the orientation of the magnetic bead. The original PMF was eliminated by respiratory inhibition with NaN_3_ in several minutes, and the stators then came off the motor on a timescale of minutes (33, 34). Thus, the rotor-hook-bead system would be undergoing rotational Brownian motion with magnetic restraint. To calibrate the stiffness of the magnetic tweezers, the bead orientation was recorded for about 360 s in magnetic constraint of suitable strength, which could be controlled by adjusting the distance between the magnets and the sample. An example bright-field image of the beads is shown in Fig. 1. The double-bead method allowed us to determine the orientation of the magnetic bead to a precision of 0.03 degree^2^ (by analyzing the angular variance of beads stuck to the glass surface), precise enough compared to the typical angular variance (4 to 6 degrees^2^) of beads attached to an inactivated motor in the magnetic tweezers.

**FIG 2:**
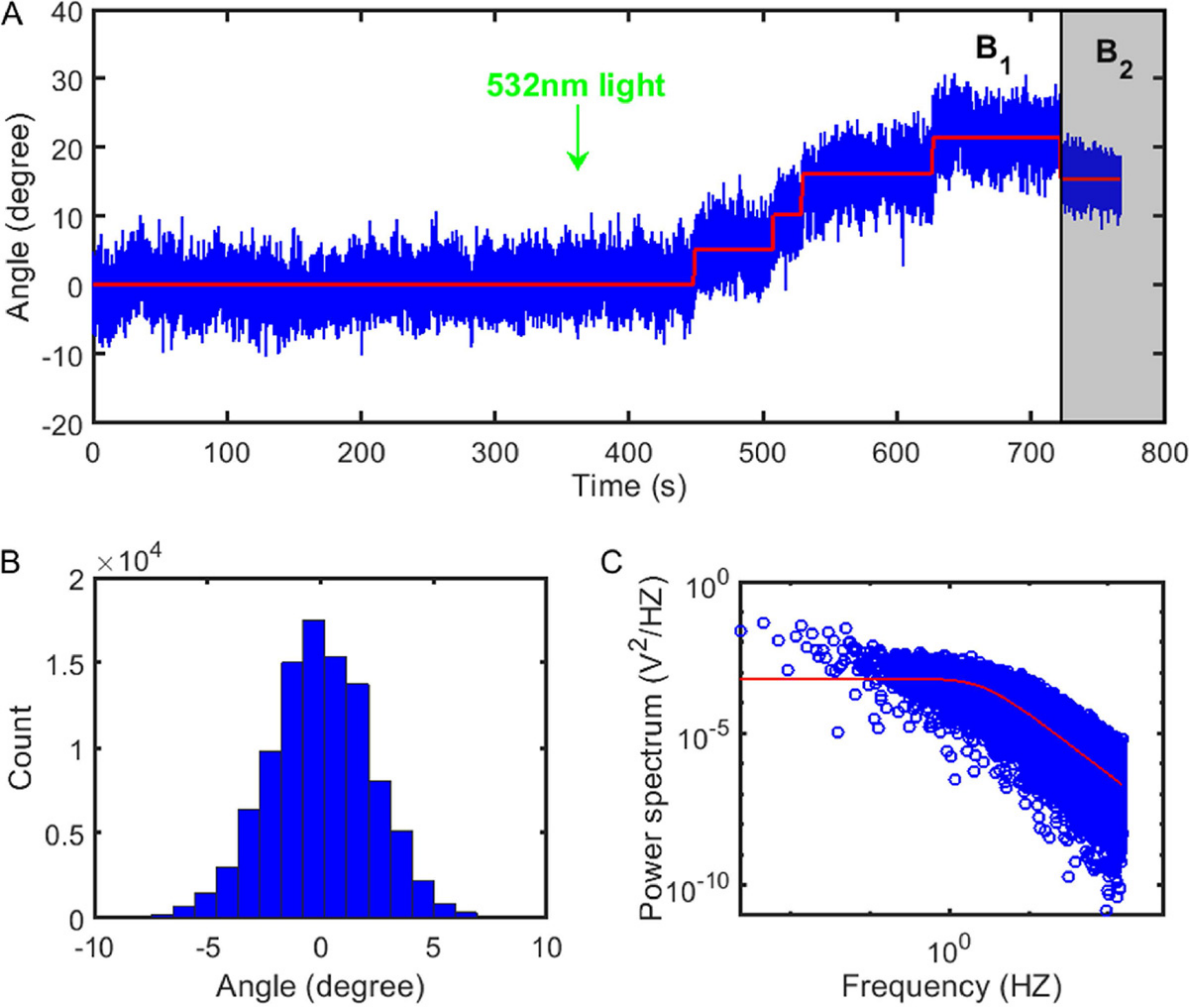
(A) A typical trace of motor resurrection at stall. The original PMF was eliminated to inactive the motor, and the magnetic bead attached to the motor was undergoing Brownian motion in the magnetic tweezers. Then 532-nm light was turned on at *t *= 360 s to restore the PMF, and the stators started to be recruited to the motor one by one, as shown in the stepping of the bead orientation angle. The magnetic field was *B*_1_ initially, and changed quickly to *B*_2_ at *t *= 720 s by moving the magnets to a closer distance from the sample with a PC-controlled translational stage. (B) Histogram of the orientation angles of the bead on the inactive motor from panel A. (C) The typical power spectrum for the trace of orientation angles of the magnetic bead when the motor was inactivated. The red line is a Lorentzian fit with the roll-off frequency extracted to be *f_c_* = 2.65 Hz.

Next, the PMF was restored by exciting the proteorhodopsin with a 532-nm laser (33–35). Consequently, the stators bound to the rotor one by one, and the orientation angle of the magnetic bead increased step by step. We recorded the motion of the bead until the angular change exceeded 30 degrees. A typical experimental trace is shown in Fig. 2A. The stiffness was extracted from the Brownian motion trace *k* = *k_B_T*/<*δθ*^2^>, where <*δθ*^2^> was the angle variance when the PMF was eliminated and the motor was inactivated (Fig. 2B), and the stall torque for each motor at each stator number was calculated from the motor resurrection trace.

### Precise calibration of the magnetic tweezers.

A crucial issue was how to precisely calibrate the torsional stiffness of the magnetic tweezers. Multiple effects, such as the bead incidentally attaching to somewhere other than the hook (e.g., the cell body), some stators still binding to the rotor, motion blur due to finite exposure time of the camera, effect due to finite frame rate of the camera, and stage drift-induced low frequency noise, might make the calibrated stiffness bigger or smaller (36, 37). To eliminate the possibility of the bead attaching to somewhere other than the hook, we performed further studies of the inactivated motors under no magnetic field, in which the rotor-hook-bead system approached free diffusive rotation, as shown in Fig. S1. If the bead was adhering to somewhere other than the hook, it is no longer free diffusion. If we calculated the stiffness with *k* = *k_B_T*/<*δθ*^2^>, assuming that the bead adhered to some linear constraint, the stiffness would be abnormally large up to 10 thousands of pN×nm. To eliminate the possibility that some stators still binding to the rotor, we did the following. It was shown previously that the PMF was restored in less than 1 s upon 532 nm light illumination (34). If some stators still bound to the rotor, the motor would immediately resurrect with the direction of the magnetic bead changed once the 532-nm laser was on; otherwise, the motor would resurrect after some period of time. Thus, we could get rid of this effect by judging whether the bead orientation immediately changed once the laser was on. Evidently, there was no stator bound to the rotor before the laser was turned on in Fig. 2A. The camera frame rate was 300 frames/s and the exposure time was 3.3 ms. The relaxation time of the magnetic bead in the tweezers, *t_relax_* = *F_θ_*/*k*, was typically about 74 ms, where *f_θ_* is the rotational frictional drag coefficient of the magnetic bead. The camera exposure time is much smaller than the relaxation time, so the motion blur was negligible (37). For a magnetic bead with rotation constrained by the magnetic tweezers, the power spectrum for its angle trace is a Lorentzian, *S*(*f*) = *A*/(1 + [*f*/*f_c_*]^2^), where *A* is a constant and *f_c_* is the roll-off frequency (see Text S1). The camera frame rate (300 fps) was far greater than *f_c_*, which was typically about 2.6 Hz (Fig. 2C), so the effect of a high-frequency cutoff due to a finite frame rate was negligible, and equivalently, the effect on the variance of the bead position was negligible according to the Parseval theorem (36). Low-frequency drift of the sample stage would add low-frequency noise to the power spectrum. To eliminate the effect of the low-frequency drift, we simulated the Brown motion of the bead trapped in the magnetic tweezers with the Langevin equation, and then we filtered the trace using a high-pass filter over a range of cutoff frequencies from 0 to 0.3 Hz. We found that the variance of the filtered angular position scaled linearly with the cutoff frequency as shown in Fig. S2. For our experimental data, the variance varied linearly with the cutoff frequency down to about 0.07 Hz, below which it was no longer linear due to drift-induced low-frequency noise (Fig. S3). Thus, we linearly fit the data between a cutoff frequency of 0.07 to 0.3 Hz and extrapolated it to 0 Hz to obtain the accurate variance of the bead angular position, as shown in Fig. S3.

10.1128/mbio.00782-22.2 FIG S1 The free rotational diffusion of the magnetic bead attached to an inactivated motor with no magnetic field. (A) The orientation angle of the bead as a function of time. (B) Mean-squared angle displacement as a function of time for the trace in panel A. Download FIG S1, TIF file, 0.5 MB. Copyright © 2022 Wang et al. 2022 Wang et al. https://creativecommons.org/licenses/by/4.0/ This content is distributed under the terms of the Creative Commons Attribution 4.0 International license.

10.1128/mbio.00782-22.3 FIG S2 Angular variance of a simulated trace (high-pass filtered) of a magnetic bead constrained by magnetic tweezers as a function of the cutoff frequency of the high-pass filter. Download FIG S2, TIF file, 0.5 MB. Copyright © 2022 Wang et al. 2022 Wang et al. https://creativecommons.org/licenses/by/4.0/ This content is distributed under the terms of the Creative Commons Attribution 4.0 International license.

10.1128/mbio.00782-22.4 FIG S3 Angular variance of an experimental trace of a magnetic bead attached to an inactivated motor in the magnetic tweezers. The trace was high-pass-filtered, and the angular variance was plotted as a function of the cutoff frequency. The red line was a linear fit to the data points above 0.07 Hz to obtain the unbiased variance by extrapolating to 0 Hz. Download FIG S3, TIF file, 0.4 MB. Copyright © 2022 Wang et al. 2022 Wang et al. https://creativecommons.org/licenses/by/4.0/ This content is distributed under the terms of the Creative Commons Attribution 4.0 International license.

### Two measurements at different magnetic strengths for individual motors.

To further ensure accuracy of the measurements of the stall torque, we performed two measurements at different magnetic strengths for each motor. The vertical distance between the magnets and the sample determines the magnetic strength for the tweezers. We selected two positions of the magnets; one corresponded to smaller strength, *B_1_*, for the magnetic tweezers (position I), and the other corresponded to the larger strength, *B_2_* (position II). At position II, the motion of the bead was recorded for about 360 s to calibrate the tweezers. The magnets were then moved to position I with the motion of the bead recorded for 360 s to calibrate the tweezers. Then the 532-nm laser was turned on to start motor resurrection. When motor resurrection proceeded long enough so that the angle change of the bead exceeded more than 30 degrees, the magnets were moved quickly to position II, reducing the angle change of the bead, as shown in Fig. 2A. We found that if the motor resurrection trace stepped stably, the relative difference for the two measurements usually satisfied:

(2) $$|\tau_{I}-\tau_{\mathrm{II}}|/(\tau_{I}+\tau_{\mathrm{II}})\leq 10\text{\%},$$

where *τ_I_* = *k*_1_ <θ_1_> and τ_II_ = *k*_2_ <θ_2_> are the motor stall torque measured when the magnets were positioned at I and II immediately before and after the position change, respectively, *k*_1_ and *k*_2_ represent the stiffness of the magnetic tweezers at position I and II, respectively, and <θ_1_> and <θ_2_> and are the angle changes (relative to the original orientation of the magnetic bead) when the magnets were positioned at I and II immediately before and after the position change, respectively. As the magnets were moved quickly from position I to position II (in less than 1 s), there was no change in stator number immediately before and after the position change of the magnets. Therefore, *τ_I_* and *τ_II_* are the measurements of the same motor stall torque at two different magnetic strengths and should be equivalent. equation 2 was a consistency check to make sure that errors in our measurements were within 10%. More examples of our experimental traces are shown in Fig. S4 and S5. Stall torques at different stator numbers were measured at magnet position I.

10.1128/mbio.00782-22.5 FIG S4 More examples of the traces of motor resurrection at stall. Download FIG S4, TIF file, 2.4 MB. Copyright © 2022 Wang et al. 2022 Wang et al. https://creativecommons.org/licenses/by/4.0/ This content is distributed under the terms of the Creative Commons Attribution 4.0 International license.

10.1128/mbio.00782-22.6 FIG S5 More examples of the traces of motor resurrection at stall. Download FIG S5, TIF file, 2.3 MB. Copyright © 2022 Wang et al. 2022 Wang et al. https://creativecommons.org/licenses/by/4.0/ This content is distributed under the terms of the Creative Commons Attribution 4.0 International license.

**FIG 3:**
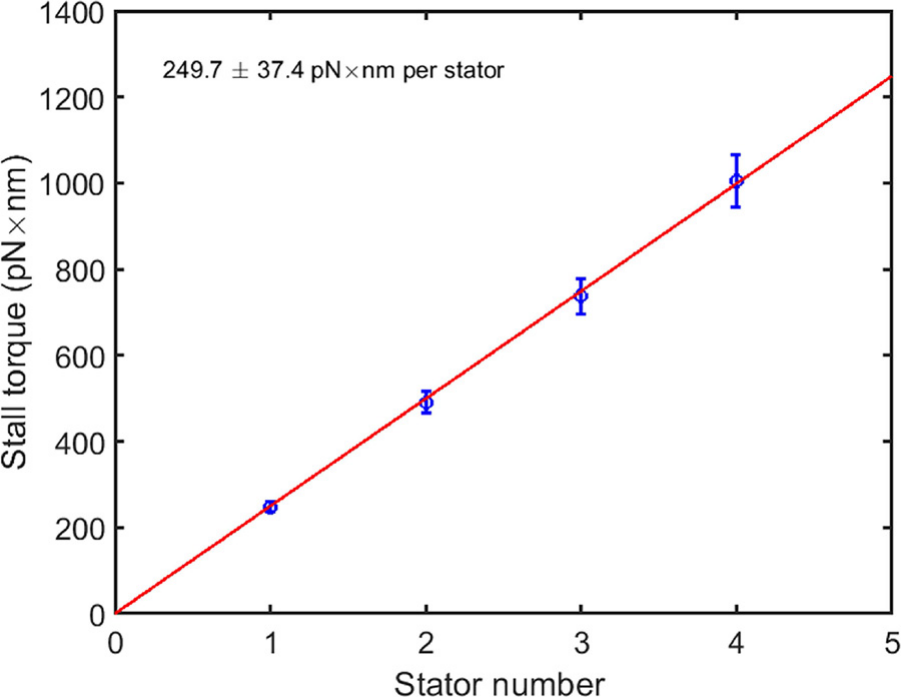
The stall torque as a function of the stator number. The data were derived from 20 motor resurrection traces at stall. The red line is a linear fit of the data to extract the stall torque per stator. Error bars are the standard error of the mean (SEM).

We measured resurrection traces for 20 motors. The average stall torques at different stator numbers are shown in Fig. 3, demonstrating a linear relationship, consistent with previous measurements at high loads (6–8, 17). This also confirmed the linearity of the magnetic tweezers. We fit the data with a linear function and extracted the stall torque per stator to be 249.7 ± 37.4 pN×nm.

Previous indirect measurements of the stall torque per stator were usually carried out with the bead assay by labeling 1.0-μm-diameter beads to the motor and assumed that the motor torque at this load is the same as the stall torque. To compare with previous measurements, we also performed motor resurrection experiments using a normal bead assay with 1.0-μm-diameter latex beads, obtaining an average value of 160.1 ± 13.6 pN×nm per stator for the motor torque at this load. Therefore, our directly measured value of the motor stall torque was about 1.56 times the motor torque under a load of 1.0-μm-diameter beads.

### Difference between the stall torque and the motor torque at high load.

**FIG 4:**
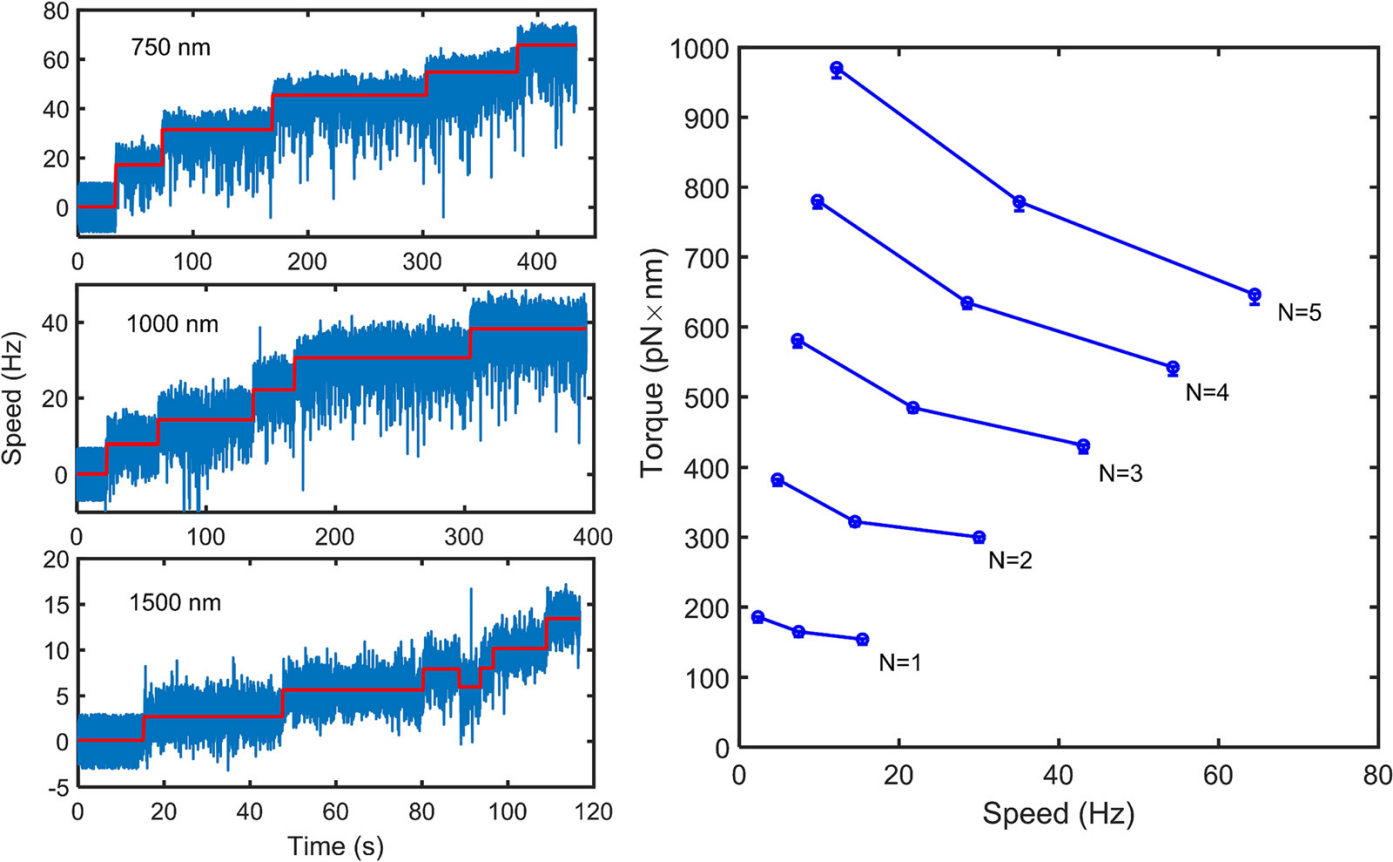
(Left panels) Typical resurrection traces for motors labeled with 0.75-, 1.0-, and 1.50-μm-diameter beads (from top to bottom). The red lines are the speed steps identified by the step-finding algorithm. (Right panel) The torque-speed curves at different stator numbers in the high-load region. From bottom to top, the stator number for each line is from 1 to 5. Data were from the motor resurrection experiments under loads of 1.5-, 1.0-, and 0.75-μm-diameter beads. The numbers of motors observed at each load were 32, 42, and 23, respectively. Error bars are the SEM.

To explore the reason behind the difference between our measured stall torque and the motor torque under a load of 1.0-μm-diameter beads, we sought to measure the torque-speed curve in the high-load region with the bead assay using different sizes of beads. In addition to the plasmid pTrc99aPR, we transformed the strain JY9 with the plasmid pKAF131, which constitutively expresses sticky filament FliC^st^. We attached 0.75-, 1.0-, or 1.5-μm-diameter beads to shortened filament stubs of the motors and carried out motor resurrection experiments using same 532-nm light conditions as the tweezer experiments. Typical resurrection traces are shown in Fig. 4 (left panels). We then constructed the torque-speed curves at different stator numbers at a high load from the resurrection traces, as shown in Fig. 4 (right panel). The motor torque at each stator number descends as the speed increases. This contributed to the difference between our directly measured stall torque and the motor torque under a load of 1.0-μm-diameter beads.

Another contribution came from the calculation of the rotational viscous drag coefficient of the load in the bead assay. The motor torque in the bead assay was calculated by multiplying this drag coefficient with the motor speed. Usually, in calculating the drag coefficient, the filament stub and the bead were assumed to be rotating in an infinitely large environment, neglecting the hydrodynamic surface effect from the cell body. As the drag coefficient came mostly from rotation of the bead, we sought to estimate the hydrodynamic surface effect on rotation of the bead. When the surface effect was neglected, the rotational drag coefficient of the bead is

(3) $$f_{b}=8\pi \eta a^{3}+6\text{πηa}r_{c}^{2},$$

where *η* is the viscosity of the medium, *a* is the radius of the bead, and *r_c_* is rotational radius of the bead. From our experiments, the rotational radius *r_c_* was about 210 nm. For simplicity, we treated the upper surface of the cell body as an infinite plane parallel to the sample glass coverslip and neglected the hydrodynamic surface effect from the glass coverslip. The rotational plane of the bead was usually not parallel to the cell body surface, with an average intersection angle of about 50 degrees. On average, the bead stuck to the filament at the length of about 1,000 nm, so the distance *s* from the center of the bead to the cell body surface was approximately 750 nm. Motion of the bead could be decomposed into two directions parallel and perpendicular to the plane of cell body. Thus, the actual rotational drag coefficient of the bead, including the surface effect is (38):

$$f_{b}^{\prime }=8\pi \eta a^{3}\sqrt{{\left(\beta^{∥}\right)}^{2}sin^{2}\theta +{\left(\beta^{⊥}\right)}^{2}cos^{2}\theta }+6\text{πηa}r_{c}^{2}\sqrt{{\left(\gamma^{∥}\right)}^{2}sin^{2}\theta +{\left(\gamma^{⊥}\right)}^{2}cos^{2}\theta },$$

where$$\gamma^{∥}=\frac{1}{1-(9/16)(a/s)+(1/8){\left(a/s\right)}^{3}},$$

$$\gamma^{⊥}=\frac{1}{1-(9/8)(a/s)+(1/2){\left(a/s\right)}^{3}},$$

$$\beta^{∥}=\frac{1}{1-(1/8){\left(a/s\right)}^{3}},$$

$$\beta^{⊥}=\frac{1}{1-(5/16){\left(a/s\right)}^{3}+(15/256){\left(a/s\right)}^{6}}.$$

Thus, $f_{b}^{\prime }\approx 1.17f_{b}$. The actual rotational drag coefficient was 1.17 times that without the surface effect as was usually done.

According to trend of the torque-speed curve in the high-load region (Fig. 4), the stall torque was ~1.22 to ~1.32 times the motor torque under the load of 1.0-μm-diameter beads, by extrapolating the torque-speed curves in Fig. 4 to stall and calculating the ratio of the resulting stall torque to the motor torque under the load of 1.0-μm-diameter beads. Combining the factor of 1.22 to ~1.32 with the factor of ~1.17 from the surface effect, the stall torque was ~1.43 to ~1.54 times the apparent motor torque under the load of 1.0-μm-diameter beads. This explains the difference between our directly measured stall torque and the apparent motor torque at high load (usually measured with 1.0-μm-diameter beads).

## DISCUSSION

During the past several decades, magnetic tweezers have widely used for studying nucleic acid enzymes (39–41). The torsional stiffness ranged from several thousands of pN · nm/rad to tens of thousands of pN · nm/rad, close to the magnitude of the stall torque of the flagellar motor. Recently, several works have applied magnetic tweezers to study the bacterial flagellar motor, finding that the magnetic field does no harm to the motor (31, 42). In this work, we took advantage of the magnetic tweezers to quantitatively measure the stall torque of the flagellar motor, which was an important parameter for modeling the motor and further understanding the working mechanism of the motor.

Here, we performed careful calibration of the magnetic tweezers by ruling out multiple possible effects. We then applied magnetic tweezers to directly measure the stall torque per stator. We made measurements of the stall torque at two magnetic strengths for each motor to verify the accuracy of our measurements. The stall torques we measured are proportional to the stator number, further confirming the linearity of the magnetic tweezers. Our directly measured stall torque is about 1.56 times the motor torque under the load of 1.0-μm-diameter beads that was previously taken to be the stall torque in indirect measurements. We explained the difference by measuring the shape of the torque-speed curve in the high-load region and by estimating the hydrodynamic surface effect from the cell body on the rotation of the bead.

We sought to estimate the number of protons, *n*, each stator passes per revolution. When the motor rotates infinitely slowly, the motor efficiency is 1, namely,

(4) $$2\pi \times T_{\mathrm{stall}}=n\times \mathrm{PMF}\times e,$$

where *T_stall_* is the stall torque per stator, and *e* is the proton charge. The PMF in a wild-type E. coli K-12 cell was about 190 mV (43). In the current study, the PMF established by the light-driven proteorhodopsin proton pump is slightly smaller. As the motor speed varies linearly with the PMF (44), we can use the ratio of the motor speeds for motors driven with wild-type PMF and motors driven with proteorhodospin-pumped PMF to extract to the latter PMF. We compared the ratio at each stator number using the motor resurrection data in this study and the wild-type motor resurrection data (10), both under the load of 1.0-μm-diameter beads, as shown in Fig. S6. The ratio is 1.12. Thus, the proteorhodopsin-pumped PMF in our experiments is about 170 mV. If we used the motor torque under the load of 1.0-μm-diameter beads measured above (160.1 pN · nm) as the stall torque as was previously normally assumed, the number of protons each stator passes per revolution would be 37, consistent with the previous measurement (24). This further confirmed that our estimate of the proteorhodopsin-pumped PMF was correct.

10.1128/mbio.00782-22.7 FIG S6 The ratio of motor speeds at each stator number for wild-type cells and cells with PMF established by light-driven proteorhodopsin. The value of the red line was 1.12 by fitting the data with a constant. Download FIG S6, TIF file, 0.2 MB. Copyright © 2022 Wang et al. 2022 Wang et al. https://creativecommons.org/licenses/by/4.0/ This content is distributed under the terms of the Creative Commons Attribution 4.0 International license.

Therefore, each stator passes 58 ± 9 protons per revolution, or equivalently, each stator passes 2.23 ± 0.34 protons per step as the motor takes 26 discrete steps per revolution (25, 26). This is consistent with the findings that each stator takes two “power strokes” per step and each power stroke is induced by one proton passing through one of the two proton channels in a stator (4, 45, 46). This supported the model that the motor rotation was tightly coupled with the proton transport (29). Based on recent cryo-electron microscopy (cryo-EM) image analyses of the purified stator complex, a gear-like rotation model between the stator and rotor was proposed (1, 2), which could explain the tight coupling mechanism.

Recent cryo-EM studies of the flagellar motor provided more information on the structure of the different rings (47–52). The FliG ring, which interacts with each stator unit, has 34-fold symmetry (47–50). The LP ring, which acts as a bushing supporting the rod for its stable rotation, has 26-fold symmetry (51, 52). These observations suggested that the flagellar motor might take 34 steps per revolution, and the previously observed 26 steps per revolution might be caused by potential minima formed by electrostatic interactions between the rod and LP ring (52, 53). If the motor took 34 steps per revolution, our measurement here would indicate that each stator passed 1.71 ± 0.26 protons per step.

Equation 4 assumed that the motor was tightly coupled with an efficiency of 1 at stall; this led to proton usage of about two per step according to our measured value of the stall torque. Thus, our measurement was consistent with the tight coupling mechanism but did not directly prove this mechanism. If the motor efficiency was less than 1, the calculated number of protons per step would be larger. To determine the actual number of protons utilized would require direct measurement of the proton flow through the motor.

## MATERIALS AND METHODS

### Strains and plasmids.

All strains for this study are derivatives of E. coli K-12 strain RP437. JY9 (Δ*cheY fliC*) carries a mutated gene, *flgE*, on the chromosome that expresses the protein with a tetracysteine motif CCXXCC at codon 220. The plasmid pTr99aPR expresses proteorhodopsin under the control of an IPTG (isopropyl-β-d-thiogalactopyranoside)-inducible promoter. The plasmid pKAF131 expresses the sticky flagellar filaments to readily adsorb polystyrene beads for the bead assay.

### Optics.

We constructed a system-combing optical trap, magnetic tweezers, 532-nm laser illumination, and bright-field imaging based on a Nikon Ti-U inverted microscope. The scheme of the setup is shown in Fig. 1. The optical trap was constructed with a 1,064-nm laser beam (AFL-1064-33-B-FA; Amonics), which was expanded 5 times with two convex lenses, reflected by a dichroic mirror (ZT1064rdc; Chroma), and focused into a diffraction-limited spot with a water-immersion objective (Nikon Plan Apo vc 60×/1.20 WI). A 532-nm fiber-coupled laser light (MGL-III-532; Cnilaser) was expanded 13 times and focused onto the back focal plane of a condenser lens by a long-focus lens. The light was reflected by a dichroic mirror (ZT543rdc-UF2; Chroma) between the condenser lens and the long-focus lens and expanded into a parallel beam by the condenser lens. There was a 1.5-mm-diameter center opening in the holder of the magnetic tweezers that allowed passage of the 532-nm laser and the bright-field illumination light. The holder was placed on a 3D personal computer (PC)-controlled motorized platform (MTS202; BeiJing Optical Century Instrument Co., Ltd.) between the condenser and the sample. The density of the 532-nm light for motor resurrection was 3.8 mW/mm^2^, at which the effect of the proteorhodopsin was saturated (Fig. S7). The light for bright-field microscopy was provided by a halogen lamp illuminating the sample from above. All convex lenses were from Thorlabs.

10.1128/mbio.00782-22.8 FIG S7 Motor speed at various intensities of the 532-nm laser. The light densities in the green zones ranging from dark to light were 3.8, 3.1, 2.3, 1.5, 0.76, and 0.38 mW/mm^2^. The light density of the other white sections was 3.8 mW/mm^2^. Download FIG S7, TIF file, 1.0 MB. Copyright © 2022 Wang et al. 2022 Wang et al. https://creativecommons.org/licenses/by/4.0/ This content is distributed under the terms of the Creative Commons Attribution 4.0 International license.

### Labeling of magnetic beads and latex beads.

Cells were grown in 3 mL of T-broth with 100 μg/mL ampicillin at 33°C to an optical density at 600 nm (OD_600_) of 0.4; then 3 μL all-transretinal and 3 μL 20 mM maleimide-PEG2-biotion (MPEGB) (33) were added, and cells were recultivated for about 1 h until the OD_600_ reached 0.5 to 0.6. Cells were harvested by washing twice with motility buffer (10 mM potassium phosphate, 0.1 mM EDTA, 10 mM lactate, and 70 mM NaCl at pH 7.0). They were mixed with 3 μL 20 mM MPEGB at 30°C for 1 h with shaking to biotinylate the hooks, rewashed with motility buffer twice, and ultimately resuspended in 300 μL motility buffer for subsequent resurrection experiments. The sample chamber was constructed by using two layers of double-sided sticky tape as a spacer between a glass slide and a glass coverslip coated with poly-l-lysine and then was placed in a baker oven at 70°C for about 10 min and subsequently allowed to cool down. Cells were flown into the chamber and allowed to stick on the coverslip in 7 min. Unstuck cells were washed away with motility buffer containing 23 mM NaN_3_, and then a solution of 2.8-μm-diameter streptavidin-coated magnetic beads (11205D; Thermo Fisher) was added into the chamber to attach spontaneously to the biotinylated hook. Unattached magnetic beads were washed away, and then a solution of 0.0015% (wt/vol) 1.0-μm-diameter biotin-labeled latex beads (F8768; Thermo Fisher) was drawn slowly into the chamber. The chamber was then sealed with Apiezon vacuum grease. The shutter for the 1,064 nm laser was opened to capture a latex bead, and the sample stage was then translated so that a streptavidin-coated magnetic bead attached to a motor was moved close to and stuck to the captured latex bead. The 1,064-nm light for the optical trap was immediately shut off. The beads were observed with bright-field microscopy, a region of interest (ROI) was chosen to cover the magnetic bead and the latex bead, and images and videos were recorded using a CMOS camera (Thorlabs; DCC1545M).

### Data analysis.

Data analysis was carried out using custom scripts in MATLAB. A latex bead was stuck to the magnetic bead as a fiducial marker to indicate its orientation, as sketched in the inset in Fig. 1. An example bright-field image of the two beads is shown in Fig. 1 (bottom right). The focusing plane was chosen so that the latex bead was in focus, and usually the magnetic bead was slightly out of focus. To calculate the angle accurately, we adapted the algorithms described in previous studies (40, 54) (see “Algorithm for Angle Detection”). Determination of angle steps in motor resurrection at stall was carried out by using a step-finding algorithm described previously (10). For the bead assay, the motor torque at different high loads was computed with the formula *T_motor_* = (*f_b_* + *f_f_*) × *ω*, where *f_b_ and f_f_* are the rotational drag coefficients of the bead and the filament stub, respectively, and *ω* is the rotational speed of the motor (23).

### Algorithm for angle detection.

The reference image that displays similar ring patterns as the magnetic bead image is

$$K(r)=K_{0}e^{-r/r_{0}}s\mathrm{in}\left(\frac{r}{\lambda }+\frac{2\pi p}{3}\right),$$

where *K*_0_ is a constant, *r* is the distance from the image center, *r*_0_ is a decay length, *λ* is the fringe spacing, and *p* determines the shift in the ring pattern (54). The values of the parameters we used were *K*_0_ = 1, *r*_0_ = 30, λ = 4, and *P* = 0.5. The reference image was convoluted with the real image to find the center of the magnetic bead at single-pixel precision (83 nm). To further get subpixel resolution, each pixel of the real image was divided into 5 × 5 subpixels, the intensities of which were obtained by linear interpolation. Then an autocorrelation calculation was performed with a shift grid of 11 × 11 around this center of the magnetic bead to get the center position at subpixel precision. A ring-shaped region (inner and outer radiuses are 15 and 35 pixels, respectively) was selected from the real image around the center of the magnetic bead that covered the image of the latex bead. It was then transformed into a polar intensity profile with linear interpolation (54), using a polar coordinate centered on the magnetic bead with the angular coordinate segmented into steps of 0.2°. The polar profile was summarized over radius *r* to obtain the angular profile:

$$p\left(\varphi \right)=\sum_{r_{\min}}^{r_{\max}}p(r,\varphi ).$$

Then *p*(*φ*) was cross correlated with *p*^–1^(*φ*), which was derived from the mirror image of the ring-shaped image about the *x* axis, to derive the shift *φ*_0_ at the highest correlation, and the orientation of the magnetic bead was

$$\pi -\frac{\varphi_{0}}{2}.$$
