# Supplementary material for: Direct Measurement of the Stall Torque of the Flagellar Motor in Escherichia coli with Magnetic Tweezers
Source: mBio. 2022 Jun 14;13(4):e00782-22. doi: 10.1128/mbio.00782-22 (PMC9426426; doi:10.1128/mbio.00782-22)
Supplement: TEXT S1 [file mbio.00782-22-s0001.docx]

**Supplemental Methods**

**Power spectrum for the rotation of the magnetic bead in magnetic tweezer**

For a magnetic bead attached to an inactivated motor in magnetic tweezer, the Langevin equation for its rotation is

$f_{\theta}\frac{d\theta(t)}{dt}+k\theta\left( t \right)=F_{therm}(t)$, (1)

where *f_θ_* is the rotational drag coefficient of the bead, *k* is the torsional stiffness of the magnetic tweezer, and $F_{therm}(t)$ was the random force due to Brown collision, with $<F_{therm}\left( t \right)>$*=* 0, ${<F}_{therm}\left( t \right)F_{therm}\left( t+\tau\right)>\propto\delta(\tau)$. In equation (1), $t$ was replaced by $t+\tau$, and then equation was multiplied by $\theta\left( t \right)$, subsequently integrated for $t$ over 0 to *T* at both sides.

$f_{\theta}\frac{1}{T}\frac{d\int_{0}^{T} \theta\left( t \right)\theta\left( t+\tau\right)dt}{d\tau}+k\frac{1}{T}\int_{0}^{T} \theta\left( t \right)\theta\left( t+\tau\right)dt=\frac{1}{T}\int_{0}^{T} \theta\left( t \right)F_{therm}\left( t+\tau\right)dt$ (2)

For $T\to\infty$ and $\tau>0$, equation (2) was transformed into:

$f_{\theta}\frac{dCorr(\tau)}{d\tau}+kCorr(\tau)=0,$ (3)

where $\mathrm{Corr}\left( \tau\right)= <\theta\left( t \right)\theta\left( t+\tau\right)> =\lim_{T\to\infty} \frac{1}{T}\int_{0}^{T} \theta\left( t \right)\theta\left( t+\tau\right)dt$ is the temporal correlation function. So the temporal correlation function had a form of exponential decay:

$Corr\left( \tau\right)=Corr(0)exp(-\frac{k}{f_{\theta}}\tau)$ (4)

The associated power spectrum was then obtained according to the Wiener-Khinchin theorem to be

$S\left( f \right)=A/{(1+{(f/f_{c})}^{2}}),$ (5)

a Lorentzian where *A* was a constant, and $f_{c}$ was the rolloff frequency with $2\pi f_{c}=k/{f_{\theta}}$.

An example of the power spectrum for the trace of a magnetic bead attached to an inactivated motor in the magnetic tweezer is shown in Fig. 2C. The Lorentzian fit gave $f_{c}$= 2.65 Hz. The stiffness was 3033.7$PN\cdot nm/rad$ from $k={k_{B}T}/{<\delta\theta^{2}>}$. So the drag coefficient *f_θ_* was 182.2 $PN\cdot nm\cdot s$, consistent with that estimated from hydrodynamic calculation.

**Linearity of the magnetic tweezer**

The torque is proportional to the sine of the angle between the magnetic field and the induced magnetic dipole (instead of the anisotropy axis of the bead). The direction of the induced magnetic dipole lies between the magnetic field and the anisotropy axis, such that the torque scales linearly with the angle *θ* between the magnetic field and the anisotropy axis for a large angular range, as explained briefly below, and experimentally verified in supplemental ref. 1.

The superparamagnetic bead is usually composed of ferrite nanoparticles dispersed in a matrix of polystyrene. The free energy of the bead in a homogeneous magnetic field was given by (supplemental ref. 2):

$F=N(\frac{1}{2}CV{sin}^{2}\varphi-BMVcos(\theta-\varphi))$, (6)

where *N* is the number of ferrite particles inside the bead, *C* is the crystalline anisotropy constant, *V* is the particle volume, $\varphi$ is the angle between the orientation of magnetization vector ***M*** and the anisotropy axis, and $\theta$ is the angle between the magnetic field ***B*** and the anisotropy axis. For a fixed value of *θ*, the orientation of magnetization vector was determined by minimizing the free energy:

$\frac{\partial F}{\partial\varphi}=0$. (7)

Thus the relation between $\theta$ and $\varphi$ was derived:

$\frac{1}{2}Csin\left( 2\varphi\right)=BMsin(\theta-\varphi)$*,*  (8)

This equation showed the dependence of $\varphi$ on $\theta$, and $\varphi=0$when $\theta$= 0. The torque of magnetic tweezers was obtained by differentiating the free energy with respect to $\theta$:

$\tau=\frac{dF}{d\theta}=NBMVsin\left( \theta-\varphi\right).$ (9)

By expanding the right-hand side of equation (9) with respect to *θ* centering on 0, and preserving up to the first-order expansion,

$\tau\approx NBMV(1-\left. \frac{d\varphi}{d\theta} \right|_{\theta=0})\theta$. (10)

Differentiating the equation (8) with respect to $\theta$, we could calculate the term $\frac{d\varphi}{d\theta}$:

$\frac{d\varphi}{d\theta}=\frac{BMcos(\theta-\varphi)}{Ccos2\varphi+BMcos(\theta-\varphi)}$.

We could continue to obtain the second-order expansion,

$\tau_{sec}=-\frac{1}{2}NBMV\left. \frac{d^{2}\varphi}{d\theta^{2}} \right|_{\theta=0}\theta^{2}$,

It was easy to see that $\left. \frac{d^{2}\varphi}{d\theta^{2}} \right|_{\theta=0}=0$ by differentiating the equation (8) again. So the second-order term is zero, and for small angle approximation,

$\tau\approx k\theta$,

where $k=NV\frac{CBM}{C+BM}$.

This linear relation was confirmed experimentally for a large angular range of *θ* in supplemental ref. 1.

**Supplemental references:**

1. Romano, G., Sacconi, L., Capitanio, M. & Pavone, F. S. Force and torque measurements using magnetic micro beads for single molecule biophysics. *Opt. Commun.* **215**, 323-331 (2003).
2. Normanno, D., Capitanio, M. & Pavone, F. S. Spin absorption, windmill, and magneto-optic effects in optical angular momentum transfer. *Phys. Rev. A.* **70**, 053829 (2004).
